# Individualized prediction of stroke-associated pneumonia for patients with acute ischemic stroke

**DOI:** 10.3389/fneur.2025.1505270

**Published:** 2025-02-07

**Authors:** Lulu Zhang, Qi Wang, Yidan Li, Qi Fang, Xiang Tang

**Affiliations:** ^1^Department of Neurology, The First Affiliated Hospital of Soochow University, Suzhou, China; ^2^Clinical Research Center, Shanghai General Hospital, Shanghai Jiao Tong University School of Medicine, Shanghai, China; ^3^Department of Biostatistics, School of Public Health, Fudan University, Shanghai, China

**Keywords:** stroke-associated pneumonia, acute ischemic stroke, prediction, easy-to-use model, SHapley Additive exPlanations

## Abstract

**Background:**

Stroke-associated pneumonia (SAP) remains a neglected area despite its high morbidity and mortality. We aimed to establish an easy-to-use model for predicting SAP.

**Methods:**

Two hundred seventy-five acute ischemic stroke (AIS) patients were enrolled, and 73 (26.55%) patients were diagnosed with SAP. *T*-test, Chi-square test and Fisher’s exact test were used to investigate the associations of patient characteristics with pneumonia and its severity, and multivariable logistic regression models were used to construct a prediction scale.

**Results:**

Three variables with the most significant associations, including age, NGT placement, and right cerebral hemisphere lesions combined with gender, were used to construct a stroke-associated pneumonia prediction scale with high accuracy (AUC = 0.93). Youden index of our SAP prediction model was 0.77. The sensitivity and specificity of our SAP prediction model were 0.89 and 0.88, respectively.

**Conclusion:**

We identified the best predictive model for SAP in AIS patients. Our study aimed to be as clinically relevant as possible, focusing on features that are routinely available. The contribution of selected variables is visually displayed through SHapley Additive exPlanations (SHAP). Our model can help to distinguish AIS patients of high-risk, provide specific management, reduce healthcare costs and prevent life-threatening complications and even death.

## Introduction

Stroke-associated pneumonia (SAP) was defined as the spectrum of lower respiratory tract infections within the first 7 days after stroke onset (1). SAP is one of the most frequent and a potentially preventable complication after acute ischemic stroke (AIS) (2, 3), which shows a consistent association with an increased risk of prolonged hospitalization (4), a high incidence of severe disability (4), mortality (5), and an associated financial burden on the medical system (6–8).

A range of factors may be associated with SAP. These include risk factors such as age (9–12), stroke severity measured by the National Institutes of Health Stroke Scale (NIHSS) (13) or the modified Rankin Scale (MRS) (14), level of consciousness, chronic obstructive pulmonary disease and coronary artery disease (11, 15, 16). Dysphagia patients are more than three times at risk of developing SAP and the risk increases 11-fold in patients with confirmed aspiration especially among those with a decrease in salivary clearance and poor oral hygiene (17). Diabetes or hyperglycemia on admission, smoking, history of pneumonia, low albumin blood level and the presence of other types of infection at the time of admission have all been found to increase the risk for SAP (18, 19). Previous research has shown associations between distinct lesion locations and SAP. Left anterior cerebral artery stroke, lesion size greater than 1/3 of the middle cerebral artery (20), brain stem infarction (21), multi-hemispheric infarction (22) and non-lacunar basal ganglia infarct (17) were demonstrated risk factors for SAP. Other risk factors include: mechanical ventilation (23), APACHE II score/organ failure status, male sex (16), atrial fibrillation (24), admission from a nursing home (25) and dysarthria (26).

To our knowledge, the incidence, predictors and outcomes of SAP after AIS have not been thoroughly reviewed among stroke units (27). Findings are inconsistent due to the comparatively small sample sizes, different dysphagia screening methods, various inclusion criteria and inaccurate classification of brain regions. Therefore, it is urgent to find a more objective, comprehensive and easily applicable model for predicting the development of pneumonia in AIS patients but these goals remain challenging in clinical practice.

In our previous research, we established a predictive model for dysphagia in AIS patients, which can predict its occurrence very early (28). The present study took advantage of comprehensive clinical data from early clinical swallowing examinations. The aims of this study were: (1) to establish a reliable predictive model of SAP based on the combined effects of multiple variables intelligently; (2) to compare the discrimination of our model and prior scores with regard to SAP after AIS; (3) to display the contribution of selected variables visually through SHapley Additive exPlanations (SHAP).

## Methods

### Study design and participants

We retrospectively identified AIS patients in the medical system of our stroke database admitted to the stroke unit between October 2017 and May 2018. The inclusion criteria were based on the diagnosis of AIS as confirmed on diffusion-weighted MRI (DWI) and was hospitalized within 48 h of the onset of stroke. Patients with the following conditions were excluded from the study: (1) preexisting pneumonia; (2) concomitant intracerebral hemorrhage; (3) brain tumors; (4) severe hepatic and renal dysfunction; (5) end-stage severe disease and (6) chronic inflammation. All subjects were divided into two groups: (1) patients with SAP; and (2) patients without SAP.

We assessed pneumonia symptoms and signs during the first 7 days of hospitalization. The modified Centers for Disease Control and Prevention (CDC) criteria were used to define SAP (29). The diagnosis of SAP required: (A) at least 1 of the following: (1) fever (≥38°C) with no other recognized cause; (2) leukopenia (≤4 × 10^9^/l) or leukocytosis (≥10 × 10^9^/l); (3) for adults ≥70 years old, altered mental status with no other recognized cause, and (B) at least 2 of the following: (1) new onset of purulent sputum, change in character of sputum over a 24 h period, increased respiratory secretions, or increased suctioning requirements; (2) new onset or worsening cough, dyspnea, or tachypnea (respiratory rate > 25/min); (3) rales, crackles, or bronchial breath sounds; (4) worsening gas exchange (e.g., O_2_ desaturation [e.g., PaO_2_/FiO_2_ ≤ 300], increased oxygen requirements) and (C) new onset or increased lung infiltrate on at least one chest X-ray (30).

Post stroke dysphagia (PSD) was assessed using the water-swallowing test (WST). The WST was performed using 30 mL of water with the patient sitting and at a 90° angle (31). Patients who presented with any signs of impaired efficacy and/or safe swallowing were considered to have PSD.

The study protocol was approved by the ethics committee of the First Affiliated Hospital of Soochow University (No. 2022210). Each participant was asked to sign two copies of informed consent; one copy was kept in the stroke center office, which was also scanned and saved in PDF format. Four separate consents were obtained: one for non-blood biomarkers, one for taking of blood samples, one for the image acquisition and another for storage of blood samples for future analyses. The ethics committee of our hospital approved the study protocol. Informed consent was obtained from all patients or their relatives upon admission.

### Imaging procedures

All patients were scanned in a 3 T MR scanner (MAGNETOM Skyra; Siemens Healthineers, Erlangen, Germany). MRI brain scans were obtained within 3 days after symptom onset for each participant admitted to the hospital. Two trained neurologists who were blinded examined all of the preprocessing results to assure the imaging quality.

### Vascular risk factors and other covariates

We collected the following variables: age, gender, medical history (including hypertension, diabetes mellitus, smoking, history of stroke, atrial fibrillation, and other heart diseases), clinical data on admission (including relevant laboratory indicators, stroke severity as measured by the NIHSS, thrombolytic/endovascular treatment, epilepsy and hospital stay), and location of stroke (left, right, bilateral hemispheres or posterior circulation). The etiology of stroke was determined according to TOAST, which refers to five classifications: (1) large-artery atherosclerosis (LAA), (2) cardioembolism (CE), (3) small vessel occlusion (SVO), (4) stroke of other determined etiology (SOE), and (5) stroke of undetermined etiology (SUE). The treating team made the diagnoses of progressive stroke. The following diagnostic criteria were used for progressive stroke: (a) the disease course extended from 6 h to 7 days; (b) the primary nervous symptoms and signs of cerebral infarction progressively worsened after regular treatment for cerebral infarction, and the NIHSS score increased no less than 2 points.

Acute Ischemic Stroke-Associated Pneumonia Score (AIS-APS) (32) was used to compare the prediction effect with our SAP prediction model.

### Statistical tests

Continuous variables are presented as the mean ± standard deviation (SD) and were analyzed using *T*-test. Categorical data were examined using the Chi-square test or Fisher’ s exact test. Multivariate logistic regression was used to construct the SAP prediction scale. The Youden index and McNemar’s test were exploited to compare the prediction effect between our SAP and AIS-APS prediction models. The Chi-square test was utilized to compare significant differences for different risk factor combinations between SAP patients and non-SAP patients. Receiver operating characteristic (ROC) analysis was applied to test the accuracy of the predictive scales.

Variables with significant associations by *T*-test, Chi-square test or Fisher’s exact test were combined with gender and used to construct the SAP prediction scale using a multivariate logistic model. The data were organized and analyzed using SAS 9.4 and R 4.2.0 software.

**Figure 1:**
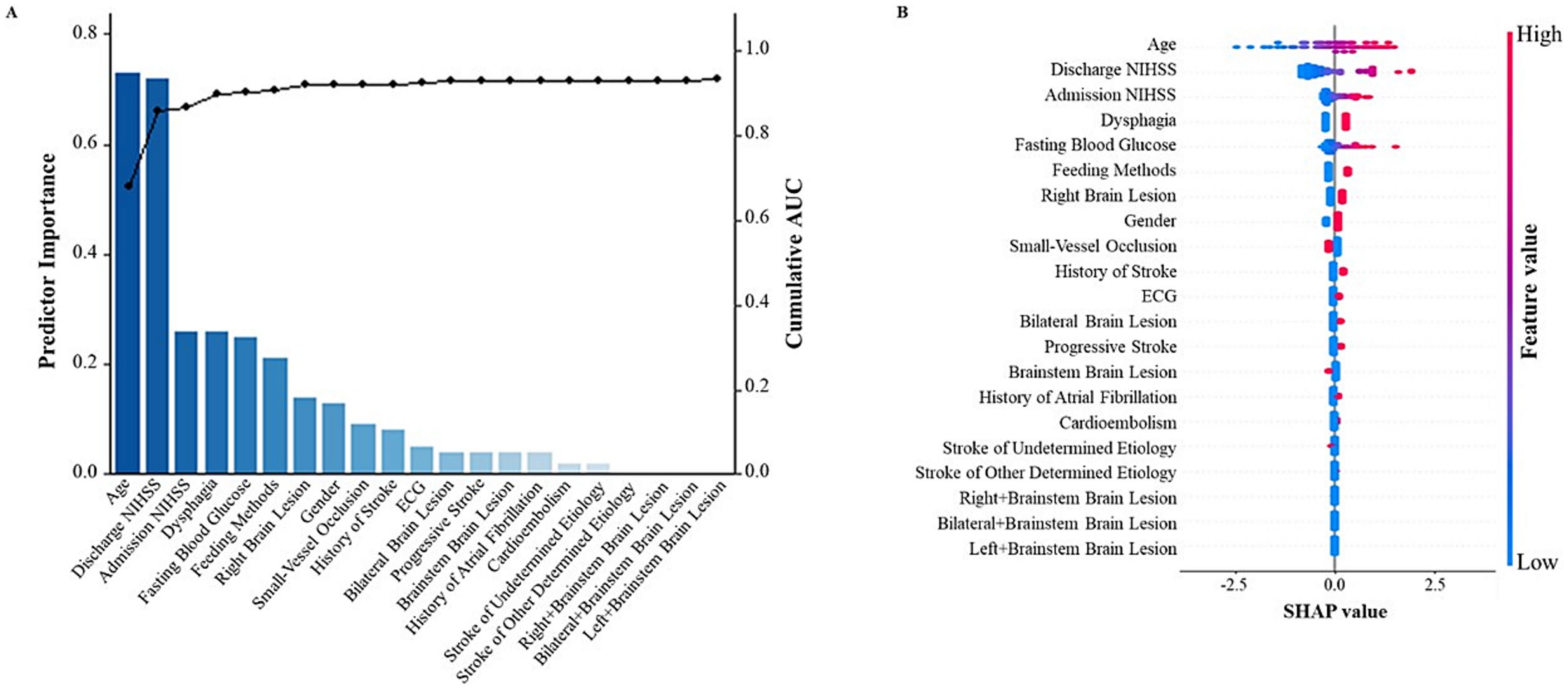
SHapley Additive exPlanations (SHAP) visualization of selected predictors modeled for all SAP patients. **(A)** The bar graph represents the contribution of the predictor to the model classification. The line graph depicts the cumulative AUCs (right axis). **(B)** The width of the range of horizontal bars can be interpreted as the impact on the model prediction with a wider range, there is a larger impact. The color of the horizontal bars represents the magnitude of predictors, which was coded in a gradient from blue (low) to red (high), shown as the color bar on the right-hand side. SHAP, SHapley Additive exPlanations.

Figure 1 was created using a combination of R 4.2.0 and Python 3.8.10. Specifically, *R* was used to generate a bar graph displaying the importance of each predictor and a line graph of the cumulative AUC values. Python was used to calculate the SHA*p* values and generate the corresponding SHAP visualization. The analysis was based on a logistic regression model, with the predictors included in the model corresponding to those used in the SAP prediction model described in the article. The SHAP visualization effectively demonstrated the impact of the predictive factors on the prediction results of our model.

## Results

A total of 275 patients fulfilled the inclusion criteria and were included in the study (Supplementary Figure S1 summarizes details of study recruitment). A total of 73 (26.55%) of the patients had SAP. The demographic and clinical characteristics of the study population and the differences between SAP and non-SAP patients are shown in Table 1. The groups significantly differed in age, and SAP patients were older than the non-SAP patients (70.74 ± 11.89 vs. 63.26 ± 12.80 years, *t* = −4.36, *p* = 1.84 × 10^−5^). SAP was associated with a higher chance of post stroke dysphagia (86.30% vs. 24.75%, *χ*^2^ = 83.92, *p* = 5.15 × 10^−20^) and nasogastric tube (NGT) placement (72.60% vs. 10.40%, *χ*^2^ = 105.50, *p* = 9.49 × 10^−25^). Patients with a history of atrial fibrillation (AF) (31.51% vs. 8.42%, *χ*^2^ = 23.00, *p* = 1.62 × 10^−6^), AF detected by Electrocardiography (ECG) (38.36% vs. 10.40%, *χ*^2^ = 28.63, *p* = 8.76 × 10^−8^), history of stroke (26.03% vs. 12.38%, *χ*^2^ = 7.44, *p* = 6.38 × 10^−3^), and higher fasting blood glucose (FBG) (6.98 ± 3.08 vs. 5.61 ± 1.84 μmoL/L, *t* = −3.57, *p* = 4.22 × 10^−4^) were more likely to suffer from dysphagia. SAP patients showed higher scores in both admission NIHSS (11.99 ± 6.66 vs. 4.25 ± 4.53, *t* = −9.19, *p* = 1.03 × 10^−17^) and discharge NIHSS (9.63 ± 6.43 vs. 3.07 ± 3.47, *t* = −8.29, *p* = 5.19 × 10^−15^). Patients with progressive stroke had a higher chance of exhibiting SAP (27.40% vs. 5.94%, *χ*^2^ = 24.01, *p* = 9.58 × 10^−7^). A strong association was found between SAP and stroke using TOAST classifications (*χ*^2^ = 49.51, *p* = 4.57 × 10^−10^). We investigated the associations of SAP and stroke using the TOAST classification as shown in Supplementary Figure S2. Patients with LAA and CE suffered SAP more often (LAA: OR = 2.20, *p* = 4.40 × 10^−3^; CE: OR = 4.61, *p* = 7.39 × 10^−7^), and patients with SVO suffered less often (OR = 0.15, *p* = 1.99 × 10^−7^). Patients of SOE and SUE did not show significant differences. Patients with or without SAP showed significant associations between distinct lesion locations (*z* = 2.88, *p* = 3.60 × 10^−3^). The associations between SAP and lesion locations are shown in Supplementary Figure S3. Patients with right cerebral hemisphere lesions suffered SAP more often (OR = 2.46, *p* = 1.22 × 10^−3^), and patients with left cerebral hemisphere lesions suffered less often (OR = 0.48, *p* = 1.54 × 10^−2^).

**Table 1:** Demographic and clinical data of patients with stroke associated pneumonia and controls.

Demographic and clinical data	Controls (*n* = 202)	Pneumonia (*n* = 73)	*t*/*χ*^2^/*z*	*p*-value
Age (years)	63.26 ± 12.80	70.74 ± 11.89	*t* = −4.36	1.84 × 10^−5^*
Gender female/male	134/68	48/25	*χ*^2^ = 0.01	0.93
Post stroke dysphagia yes/no	50/152	63/10	*χ*^2^ = 83.92	5.15 × 10^−20^**
Nasogastric tube placement yes/no	21/181	53/20	*χ*^2^ = 105.50	9.49 × 10^−25^**
Systolic blood pressure (mmHg)	144.30 ± 22.05	142.50 ± 18.49	*t* = 0.63	0.53
Diastolic blood pressure (mmHg)	81.27 ± 13.20	79.88 ± 12.30	*t* = 0.78	0.43
History of hypertension yes/no	143/59	58/15	*χ*^2^ = 2.04	0.15
History of diabetes yes/no	39/163	18/55	*χ*^2^ = 0.93	0.33
Smoking yes/quit/no	52/3/147	17/5/51	*χ*^2^ = 5.50	0.06
History of AF^†^ yes/no	17/185	23/50	*χ*^2^ = 23.00	1.62 × 10^−6^*
Other heart diseases yes/no	7/195	4/69	*χ*^2^ = 0.16	0.69
Previous stroke yes/no	25/177	19/54	*χ*^2^ = 7.44	6.38 × 10^−3^*
Triglyceride (mmol/L)	1.44 ± 0.89	1.23 ± 0.51	*t* = 2.44	0.02
Total cholesterol (mmol/L)	4.27 ± 1.06	4.09 ± 1.02	*t* = 1.24	0.22
LDLC (mmol/L)	2.43 ± 0.83	2.32 ± 0.77	*t* = 0.96	0.34
Creatinine (μmol/L)	69.30 ± 18.31	70.84 ± 18.58	*t* = −0.61	0.54
Uric acid (μmol/L)	294.10 ± 84.21	289.30 ± 110.20	*t* = 0.34	0.73
Fasting blood glucose (μmol/L)	5.61 ± 1.84	6.98 ± 3.08	*t* = −3.57	4.22 × 10^−4^*
Homocysteine (μmol/L)	12.30 ± 9.25	11.23 ± 3.16	*t* = 1.43	0.15
Hemoglobin A1c (%)	6.76 ± 1.46	6.89 ± 1.45	*t* = −0.68	0.50
ECG^†^ (AF) yes/no	21/181	28/45	*χ*^2^ = 28.63	8.76 × 10^−8^**
Admission NIH stroke scale	4.25 ± 4.53	11.99 ± 6.66	*t* = −9.19	1.03 × 10^−17^**
Discharge NIH stroke scale	3.07 ± 3.47	9.63 ± 6.43	*t* = −8.29	5.19 × 10^−15^**
TOAST classification^†^	62/24/91/11/14	36/28/8/1/0	*χ*^2^ = 49.51	4.57 × 10^−10^**
Progressive stroke yes/no	12/192	20/53	*χ*^2^ = 24.01	9.58 × 10^−7^**
Brain lesion location^†^	85/55/22/35/0/3/2	19/35/9/6/1/1/2	*z* = 2.88	3.60 × 10^−3^*
Special treatment thrombolytic/endovascular/no	25/12/165	12/3/58	*z* = 0.74	0.46
Epilepsy yes/no	5/197	2/71	*χ*^2^ < 0.01	1.00
Length of hospital stay	11.62 ± 6.54	10.85 ± 5.01	*t* = 1.03	0.30

*Continuous data are shown as mean ± SD, values in patients with stroke associated pneumonia and controls with statistical significance based on two sample *T*-test. Categorical data differences in patients and controls are represented with statistical significance based on Chi-square test (*χ*^2^ and *P*) or Fisher’s exact test (*z* and *P*). *p < 0.005, **p < 1.00 × 10^−6^.

^†^AF refers to atrial fibrillation, LDLC refers to low density lipoprotein cholesterol, ECG refers to Electrocardiogram. TOAST refers to five classifications: (1) large-artery atherosclerosis, (2) cardioembolism, (3) small-vessel occlusion, (4) stroke of other determined etiology and (5) stroke of undetermined etiology. Brain lesion locations refer to seven classifications: (1) left cerebral hemisphere lesion, (2) right cerebral hemisphere lesion, (3) bilateral cerebral hemisphere lesions, (4) brainstem lesion, (5) left cerebral hemisphere and brainstem lesions, (6) right cerebral hemisphere and brainstem lesions, (7) bilateral cerebral hemisphere and brainstem lesions.

### Risk factors and prediction scale of SAP

Variables with significant associations were combined with gender and used to construct the SAP prediction scale using a multivariate logistic model. As shown in Table 2, age (OR = 1.05, *χ*^2^ = 4.00, *p* = 4.55 × 10^−2^), NGT (OR = 4.03, *χ*^2^ = 5.87, *p* = 0.02) and right cerebral hemisphere lesions (OR = 3.77, *χ*^2^ = 6.75, *p* = 0.01) showed the most significant associations with SAP in multivariate statistical analysis, and the other variables, including gender, swallowing function, history of AF, history of stroke, FBG, NIHSS score, TOAST classifications and progressive stroke, showed no association.

**Table 2:** Multivariable logistic regression model for predicting patients with pneumonia.

Variables	Odds ratio	95% CI	*χ* ^2^	*P-*value
Age	1.05	1.00, 1.10	4.00	4.55 × 10^−2^*
Gender	2.34	0.83, 6.60	2.58	0.12
Post stroke dysphagia yes/no	2.29	0.71, 7.39	1.91	0.17
Nasogastric tube placement yes/no	4.03	1.30, 12.43	5.87	0.02*
History of AF^†^ yes/no	1.78	0.35, 9.13	0.48	0.49
History of stroke yes/no	1.94	0.71, 5.31	1.66	0.20
Fasting blood glucose (μmol/L)	1.12	0.93, 1.35	1.39	0.24
ECG^†^ (AF) yes/no	5.22	0.20, 137.44	0.98	0.32
Admission NIH stroke scale	1.06	0.93, 1.22	0.78	0.38
Discharge NIH stroke scale	1.11	0.92, 1.33	1.20	0.27
Large-artery atherosclerosis	1.00			
Cardioembolism	0.23	0.01, 6.09	0.78	0.38
Small-vessel occlusion	0.57	0.18, 1.78	0.94	0.33
Stroke of other determined etiology	1.88	0.15, 23.72	0.24	0.63
Stroke of undetermined etiology	<0.01	<0.01, >999.99	<0.01	0.97
Progressive stroke	2.10	0.64, 6.92	1.48	0.22
Lesioned hemi left	1.00			
Lesioned hemi right	3.77	1.39, 10.27	6.75	0.01*
Lesioned hemi both	3.51	0.75, 16.36	2.56	0.11
Lesioned brainstem	1.19	0.25, 5.60	0.05	0.83
Lesioned hemi left and brainstem	>999.99	<0.01, >999.99	<0.01	0.99
Lesioned hemi right and brainstem	2.14	0.02, 209.00	0.11	0.74
Lesioned hemi both and brainstem	2.20	0.19, 25.10	0.40	0.53

*P < 0.05.

**Figure 2:**
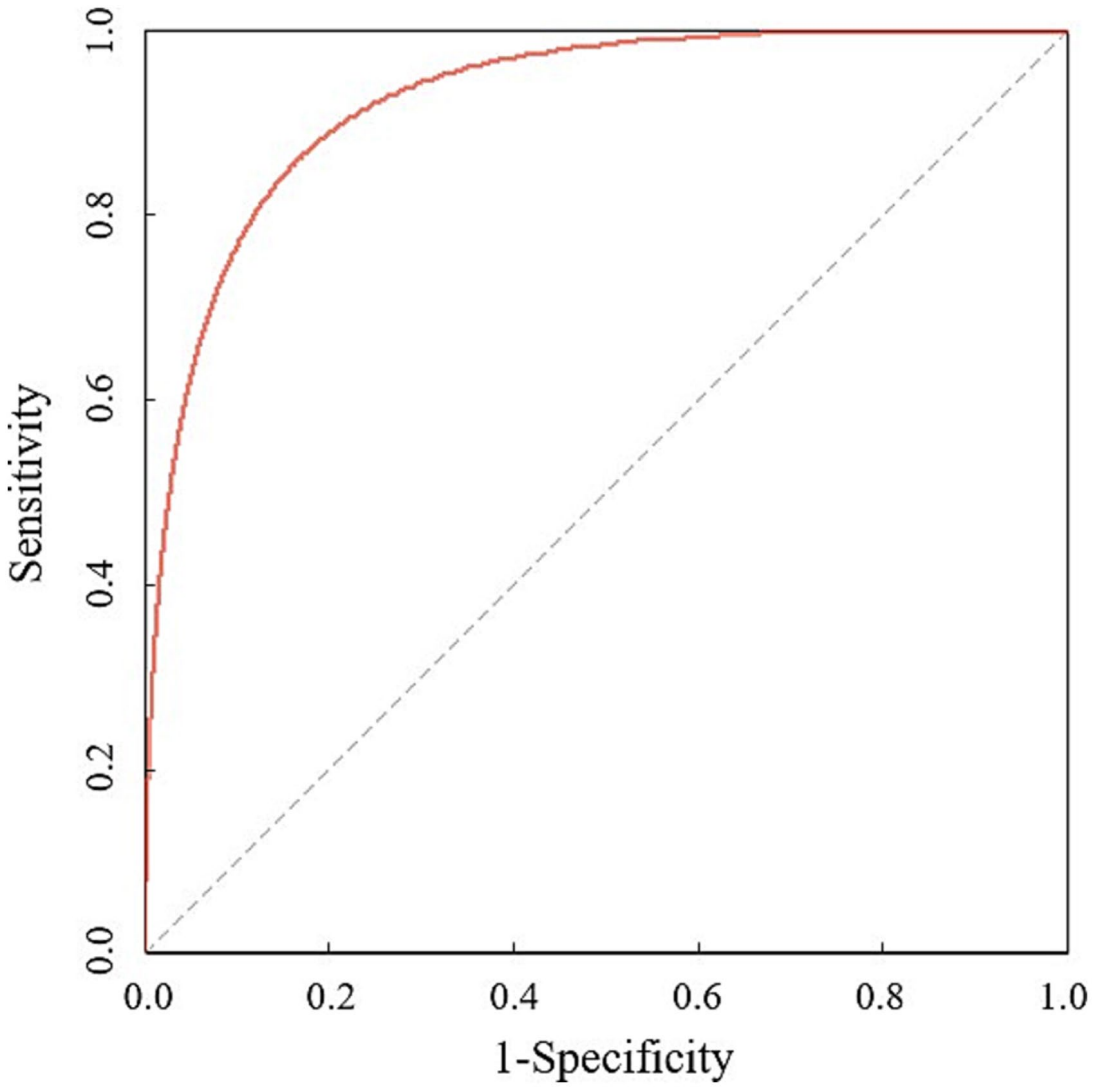
Receiver operating characteristic (ROC) curves generated for stroke with pneumonia and pneumonia severity receiver operating characteristic curve was generated for age, nasogastric tube placement and right cerebral hemisphere lesions (age and gender effects included) with stroke associated pneumonia (AUC = 0.93, *p* < 0.05) based on multiple logistic regression.

A ROC analysis was performed to examine the accuracy of the SAP prediction scale. As shown in Figure 2, age, nasal feeding diet and right cerebral hemisphere lesions together showed a significantly high AUC (area under the ROC curve) of 0.93, with *p* < 0.05. Age and gender effects were included in the multivariate logistic model to construct prediction scales.

The calculated Youden index of AIS-APS score prediction system was 0.32. According to the ROC analysis of our SAP prediction model established in this study, the best critical point was obtained, and the calculated Youden index was 0.77, which was better than that of AIS-APS score prediction system. The results of McNemar’s test showed that the sensitivity of our SAP prediction model was higher (*χ*^2^ = 39.00, *p* < 0.05), and the specificity of AIS-APS score was higher (*χ*^2^ = 15.21, *p* < 0.05).

**Figure 3:**
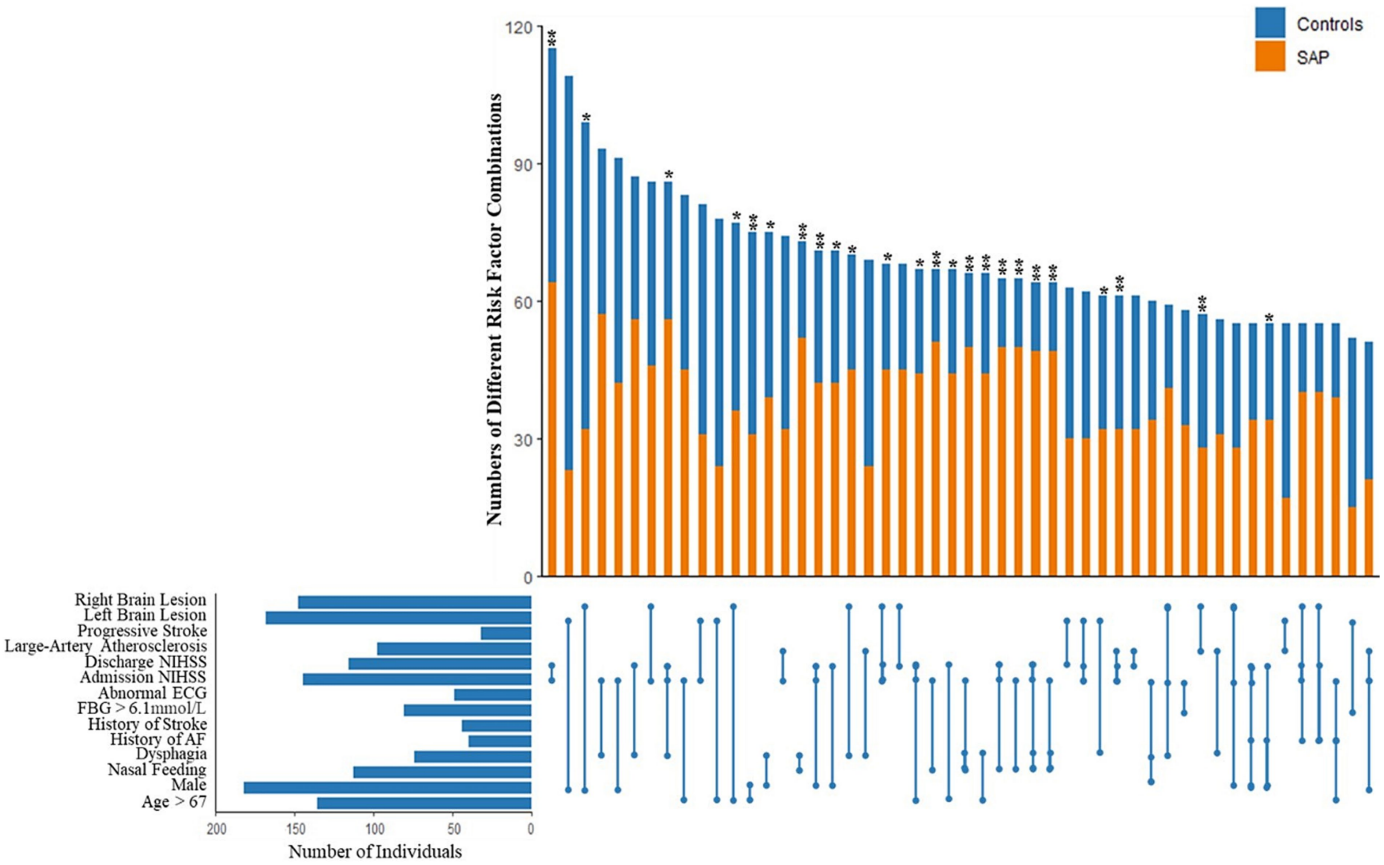
Combinations of different stroke associated pneumonia risk factors based on the 13 variables in our SAP prediction model, statistical analysis was performed on patients with 2 or more risk factors at the same time. The 50 most common combinations are shown and compared between SAP patients and non-SAP patients. After adjustment for other risk factors that were not included in the combinations, 24 combinations remained statistically significant (*p* < 0.05). OR value of each combination was calculated, and 5 combinations with the highest OR value were abnormal swallowing function, NGT, admission NIHSS >3 score and discharge NIHSS >3 score. *p < 0.05, **p < 0.01.

Based on the 13 variables in our SAP prediction model, statistical analysis was performed on patients with 2 or more risk factors at the same time. The 50 most common combinations are shown and were compared between SAP patients and non-SAP patients in Figure 3. Continuous variables were divided into 2 categories. Age was divided into two groups according to the median: ≤67 years and >67 years. FBG was divided into ≤6.1 mmoL/L and >6.1 mmoL/L. Admission NIHSS and discharge NIHSS were divided into ≤3 and >3 score. To avoid repetition, only LAA type was retained in the TOAST classification. Lesions were divided into two variables: left and right brain lesions, and patients with both left and right brain lesions were regarded as having bilateral lesions. Of the 50 combinations, the distribution of 44 combinations was significantly different between SAP patients and non-SAP patients (*p* < 0.05). After adjustment for other risk factors that were not included in the combinations, 24 combinations remained statistically significant (*p* < 0.05). OR value of each combination was calculated, and 5 combinations with the highest OR value were abnormal swallowing function, NGT, admission NIHSS >3 score and discharge NIHSS >3 score.

Once the predictive variables were determined, SHAP visualization of the selected predictors was modeled for all SAP patients as shown in Figure 1.

## Discussion

Our study demonstrated three major findings in AIS patients: (1) the potential clinical risk factors for pneumonia following AIS patients, included older age, having post stroke dysphagia, NGT placement, atrial fibrillation, history of stroke, higher fasting blood glucose, NIH stroke scale, TOAST classification, progressive stroke and brain lesions. (2) The sensitivity and specificity of our SAP prediction model were 0.89 and 0.88, respectively, focusing on features that are routinely available, and (3) SHAP visualization of selected predictors was modeled for all SAP patients.

The SAP is a complex disease and is prevalent in hospitalized patients. Occurrence of pneumonia in the early phase of stroke may promote stroke-related disability and mortality in AIS patients (4, 9). The overall incidence of SAP among majority of studies ranged from 3.90 to 56.70%, depending on the small sample size and heterogeneity of studies (33). The prevalence of SAP (26.55%) in our study is consistent with other studies in which the same diagnostic criteria were used. Various factors, such as dysphagia, aspiration, and immunodepression, are assumed to play a role in SAP. However, there remain a large number of unanswered questions (34). Understanding risk factors and determining predictors of SAP are vital in early intervention and prevention of SAP (29). We observed that SAP was significantly associated with older age, higher NIHSS on admission, dysphagia on admission, and history of atrial fibrillation, and led to more frequent mechanical ventilation and death (35).

Multiple studies (10, 36, 37) have reported that advanced age is associated with an increased risk of SAP. In this study, elderly patients were also found to be a predictor for SAP. This may be due to comorbid conditions and decreased reflex mechanisms associated with advanced age (38). Atrial fibrillation elevates the risk of cardiac embolism stroke in patients, usually leading to the obstruction of major intracranial blood vessels and resulting in a large infarction area. This process may recur repeatedly, impacting various vascular areas and resulting in severe neurological deficits, and finally increasing the risk of SAP (39). Consistent with past reports, we suggest an association between SAP and previous stroke (40). Our study supports previous studies that we found stroke-associated pneumonia to be associated with hyperglycemia (41) and stroke severity measured by NIHSS at the time of admission (9, 42). This observation indicated that the severity of stroke is higher in patients with SAP and more attention is required for SAP in severe AIS patients. Previous research had established a significant correlation between NIHSS score and the risk of developing SAP. Specifically, it had been observed that individuals with elevated NIHSS scores face an increased probability of experiencing SAP. This association can be attributed to the fact that patients who score higher on the NIHSS typically exhibit diminished levels of consciousness or are required to remain in bed for extended periods. Such conditions predisposed these patients to gastroesophageal reflux, a risk factor that significantly contributes to the development of aspiration pneumonia (43). Our research showed that patients with LAA and CE were more likely to suffer from SAP. To date, no studies have focused on the relationship between TOAST classification and SAP. One potential explanation is that patients with LAA and CE tend to have severe neurological deficits. Few advances have been made in elucidating the association between progressive stroke and SAP. Our results suggest that progressive stroke can have a significant impact on increasing the risk of pneumonia in these patients. There is increased susceptibility in the acute phase for patients with progressive stroke.

Dysphagia increased vulnerability to SAP in the acute phase after stroke as shown in previous studies (44). There is a clear correlation between dysphagia and aspiration. SAP is thought to be secondary to aspiration (9). Many dysphagic patients after stroke may suffer from aspiration of oral content especially during sleep. This phenomenon may be related to abnormal dopamine transmission (45). Experimental evidence showed that blocking D1 dopamine receptors could result in inhibition of the swallowing reflex and a decrease in substance P in end organs (46, 47). Early dysphagia screening is associated with a lower incidence of pneumonia. Guidelines support that all AIS patients should undergo standardized dysphagia screening at admission as soon as possible before oral intake, and later assessments should be performed in patients with altered consciousness (48). Delays in the assessment of swallowing were associated with an absolute risk of SAP incidence of 1% per day of delay (29).

Previous research indicated that the extended use of nasogastric tubes was linked to a higher occurrence of pneumonia and a deterioration in the outcomes for patients who had suffered a stroke (49). NGT placement contributed to pneumonia by promoting bacterial colonization due to formation of biofilms on the tube, which predisposed patients to gastroesophageal reflux and vomiting. Aspiration of bacteria laden secretions and infected refluxed material increased the risk of pneumonia. Additionally, patients with NGT usually had more severe strokes with impaired consciousness (2). Mortality and morbidity are much higher in patients with NGT which can predict the higher rate of SAP in this subset of the population (4, 18, 50, 51). The use of nasogastric tube intervention serves as a more precise and responsive measure compared to dysphagia, as it can indicate both dysphagia and disturbances in consciousness. Furthermore, while nasogastric tube intervention is recognized as a significant risk factor for SAP, existing prediction models fail to account for this critical variable. Consequently, a notable strength of our SAP prediction model lies in its inclusion of this important variable (52).

At present, only a few numbers of articles examined the influence and underlying mechanisms of the left and right cerebral hemispheres on SAP. In a model of intracerebral hemorrhage used for predicting and investigating SAP, the following observations were made: The distribution indicated a left–right imbalance correlated with the severity of SAP. Among patients experiencing severe pneumonia, over 70% exhibited hemorrhagic lesions in the left cerebral hemisphere. In those with moderate pneumonia, more than 50% presented with hemorrhagic clots located in the right side of the brain. Conversely, no significant imbalance in the distribution of the left and right cerebral hemispheres was noted in patients suffering from mild pneumonia. Given that the left hemisphere typically serves as the dominant hemisphere, its involvement was associated with more severe disease, compounded by impaired consciousness, difficulties in swallowing, and an inability to effectively expel sputum, leading to poor airway protection and severe SAP (53). It was reported that the cortical regulation of sympathetic nervous system activation was predominantly associated with the right cerebral hemisphere, whereas the activation of the parasympathetic nervous system was primarily linked to the left cerebral hemisphere (54). The activation of the sympathetic nervous system may play a significant role in immune regulation during SAP.

Our study has several limitations that need to be acknowledged. First, this was a retrospective study that included patients from a single tertiary medical center, which could have resulted in selection bias. Future research should definitely add a prospective validation to our retrospective analysis. Second, the cohort included ischemic strokes only, therefore, we cannot comment on the application of these scores to patients with hemorrhagic stroke. Third, our article only distinguishes hemispheric and brainstem lesions without identifying the association between the location and SAP based on anatomical and functional neuroimaging. Moreover, further researches are required to investigate the mechanisms involved in the pathophysiological, pathogen and changes in gut microbiota of SAP in AIS patients, which are likely to play important roles. We will pay particular attention to this point in future research.

## Conclusion

In conclusion, we analyze the effects of a comprehensive range of features for SAP in AIS patients and finally identifies the best predictive model. The result suggests that early screening for diet and brain lesions, as well as age, allows for the identification of patients at high risk for SAP. Our study was designed to be as clinically relevant as possible, focusing on features that are routinely available or could be quickly determined by simple testing. In addition, the easy-to-use prediction model we established is accurate, with a high AUC value. More than that, our study analyzes the effects of multiple combinations of risk factors for SAP in AIS patients and the contribution of selected variables is visually displayed through SHAP. SAP prediction model we established can help clinicians to distinguish high-risk AIS patients, provide specific management, reduce healthcare costs and prevent life-threatening complications and even death.

## Data Availability

The original contributions presented in the study are included in the article/Supplementary material, further inquiries can be directed to the corresponding author.
