# Supplementary material for: Individualized prediction of stroke-associated pneumonia for patients with acute ischemic stroke
Source: Front Neurol. 2025 Feb 7;16:1505270. doi: 10.3389/fneur.2025.1505270 (PMC11843556; doi:10.3389/fneur.2025.1505270)
Supplement: Supplementary file 1 [file Table_1.DOCX]

Figure S1. Details of study recruitment.

AIS: acute ischemic stroke; TIA: transient ischemic attack.

Study population (n=275)

Hemorrhagic stroke (n=45),

TIA (n=20)

Swallowing assessment

＞ 24 hours (n=21)

Possible preexisting dysphagia (n=15)

Severe hepatic and renal dysfuntion, End-stage severe disease (n=90)

Severe hepatic and renal dysfuntion (n=67)

MRI not available (n=86)

All acute stroke patients between October2017 and May 2018 from stroke unit at First Affiliated Hospital of Soochow University (n=552)

Swallowing assessment

within 24 hours (n=466)

AIS patients (n=487)
