# Supplementary figures and images for: Individualized prediction of stroke-associated pneumonia for patients with acute ischemic stroke
Source: Front Neurol. 2025 Feb 7;16:1505270. doi: 10.3389/fneur.2025.1505270 (PMC11843556; doi:10.3389/fneur.2025.1505270)

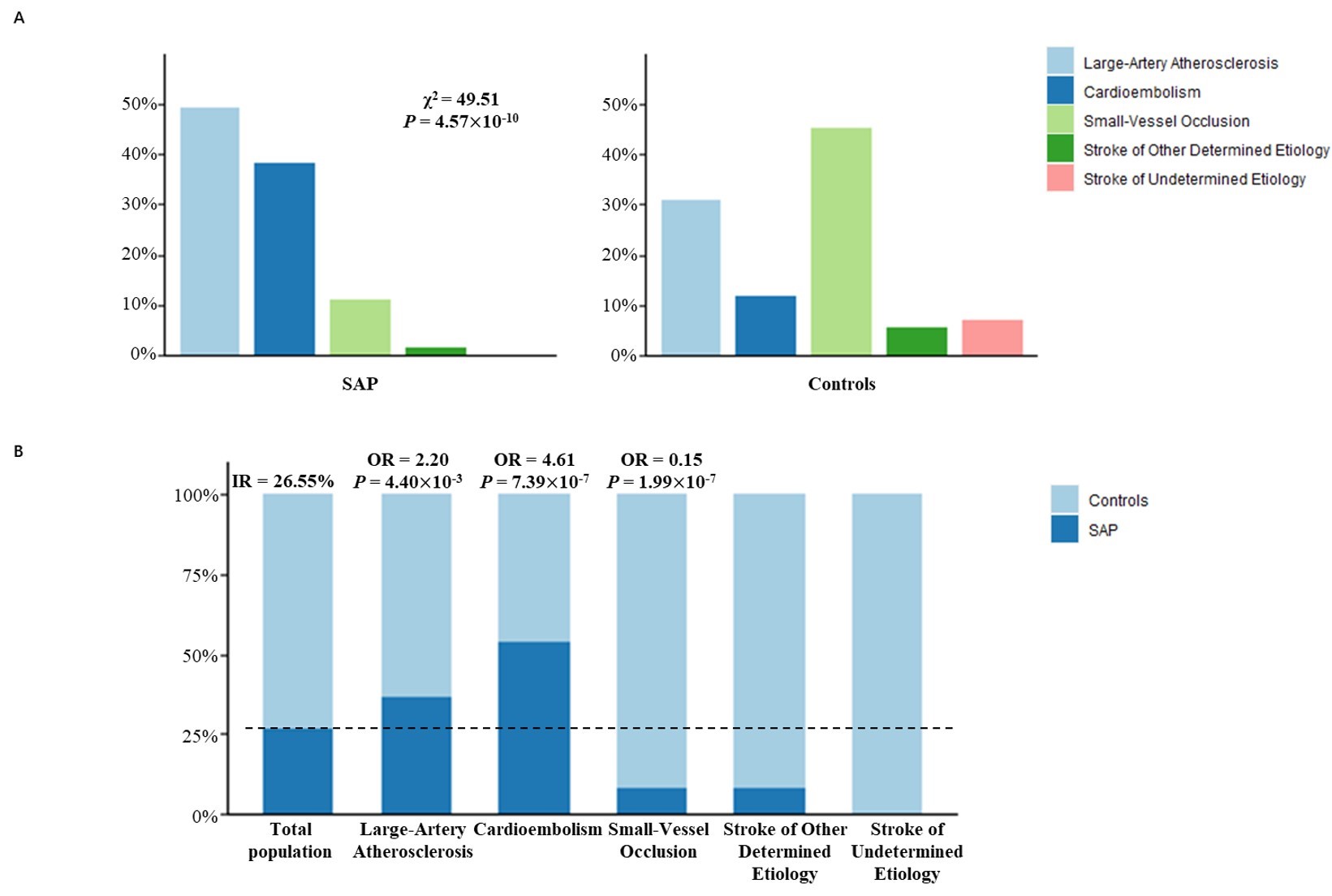

Supplement: Supplementary file 2 [file Image_1.JPEG]

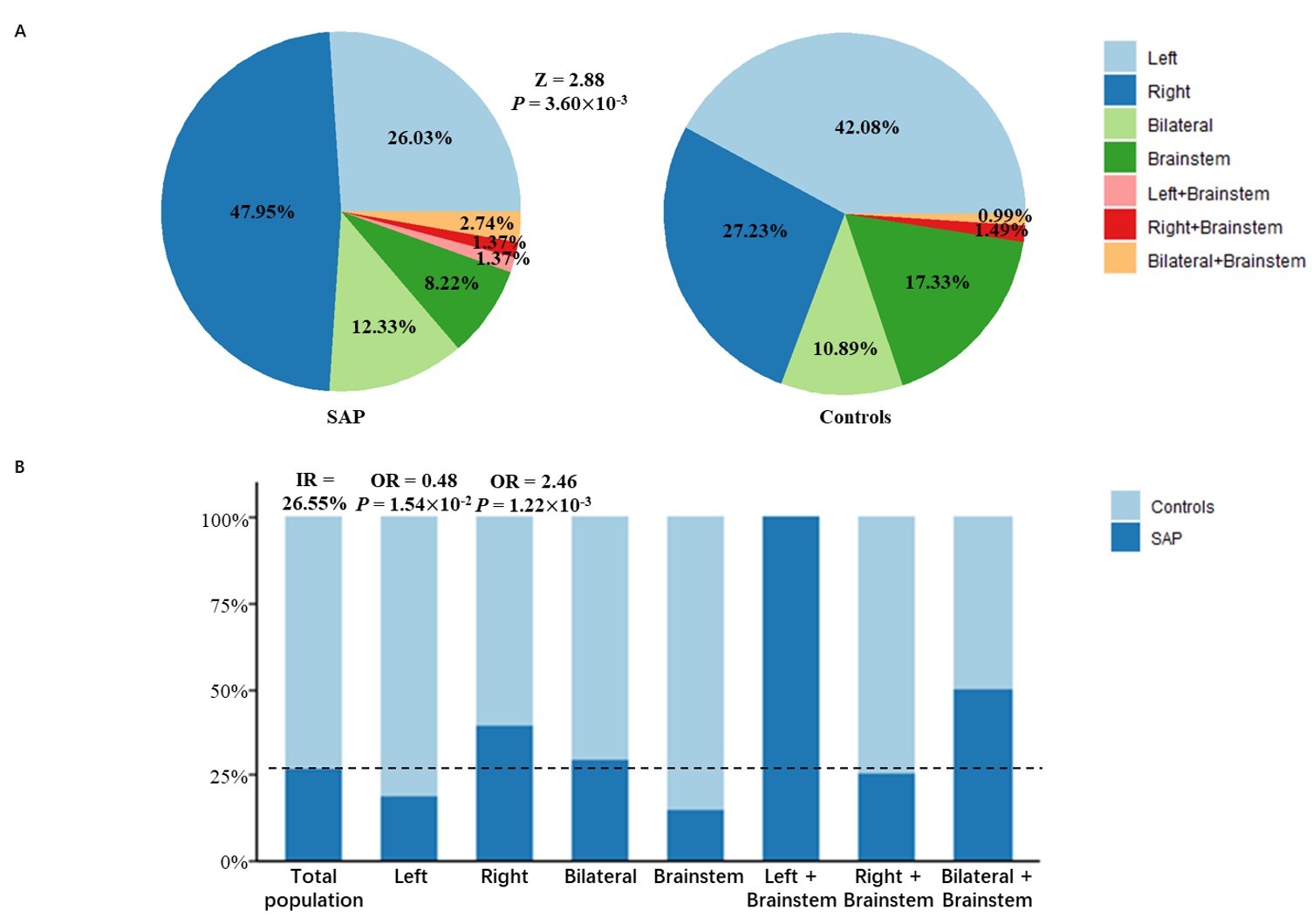

Supplement: Supplementary file 3 [file Image_2.JPEG]
